# Metastatic Pleural Effusion: An Unusual Presentation of Urothelial Bladder Carcinoma

**DOI:** 10.7759/cureus.4619

**Published:** 2019-05-08

**Authors:** Isma N Javed, Tony Abdo, Nazir Ahmad, Kellie R Jones

**Affiliations:** 1 Internal Medicine, University of Oklahoma Health Sciences Center, Oklahoma, USA; 2 Pulmonology, University of Oklahoma Health Sciences Center, Oklahoma, USA; 3 Internal Medicine, Saint Anthony Hospital, Oklahoma, USA

**Keywords:** pleural effusion, metastatic pleural effusion, urothelial carcinoma, bladder cancer, recurrent pleural effusion, pleural metastases, painless hematuria, shortness of breath

## Abstract

Pleural effusions are frequently encountered in clinical practice. In the United States, malignancy is the third leading cause of pleural effusion after heart failure and pneumonia. The most common cause of malignant pleural effusion (MPE) is lung cancer, followed by breast cancer, lymphoma, and mesothelioma. Genitourinary cancers rarely metastasize to the pleura. Although several atypical patterns of thoracic metastasis from genitourinary cancers have been described in the literature, genitourinary cancers rarely give rise to MPEs. We describe a case where the workup of a unilateral pleural effusion led to the diagnosis of high-grade urothelial bladder carcinoma.

## Introduction

Urothelial bladder cancer is the ninth most common cause of cancer worldwide [1]. It affects more men than women. Cigarette smoking is an important risk factor [2]. It can cause localized, locally invasive or metastatic disease. The most frequent sites of metastases are regional lymph nodes (90%), liver (47%), lung (45%), bone (32%), peritoneum (19%), pleura (16%), kidney (14%), adrenal gland (14%), and the intestine (13%) [3]. Typically, thoracic metastases occur in the form of pulmonary nodules. On rare occasions, urothelial bladder cancer may cause symptomatic pleural effusion drawing clinical attention towards the underlying cancer [4].

## Case presentation

**Figure 1:**
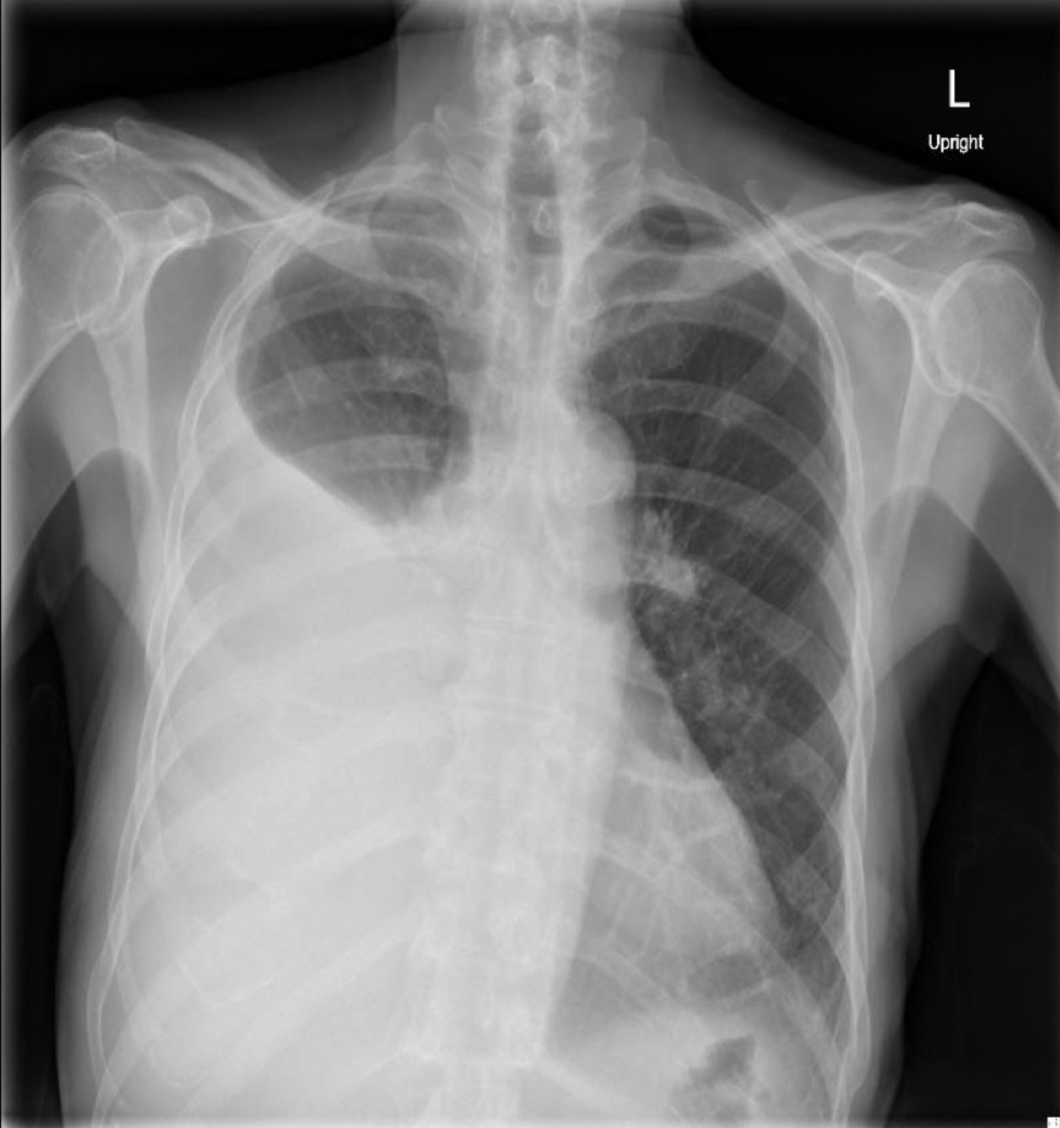
Chest radiograph (anterior-posterior projection) showing large right-sided pleural effusion

A 69-year-old man who was a heavy smoker presented to the emergency department (ED) with worsening shortness of breath. His medical history was significant for well-controlled hypertension, chronic kidney disease stage III, and right solitary kidney from a left-sided nephrectomy for atrophic kidney from ureteropelvic junction obstruction. He reported feeling fine at his baseline until one week prior to presentation. He could walk miles earlier but now would become short of breath upon walking just a few feet. He denied any documented fever, night sweats, cough, hemoptysis or chest pain. Upon further inquiry, he also reported feeling bloated. He denied experiencing similar symptoms in the past. His outpatient medications included atenolol, allopurinol, atorvastatin and over the counter ant-acids and laxatives. He had normal vital signs with normal oxygen saturation on room air. Physical exam was notable for decreased tactile fremitus, and dullness upon percussion with reduced breath sounds on the right side. Routine lab work was within normal limits. The chest X-ray showed a large right-sided pleural effusion (Figure 1). 

He was admitted under observation status. An ultrasound-guided bedside thoracentesis was performed and yielded 1.5 liters of turbid orange exudative fluid. The pleural fluid was sent for chemical analysis and cytology. The patient improved symptomatically overnight and requested to be discharged home the very next day. He was sent home with a plan to follow up cytology results. The patient’s primary care physician was notified as well.

**Figure 2:**
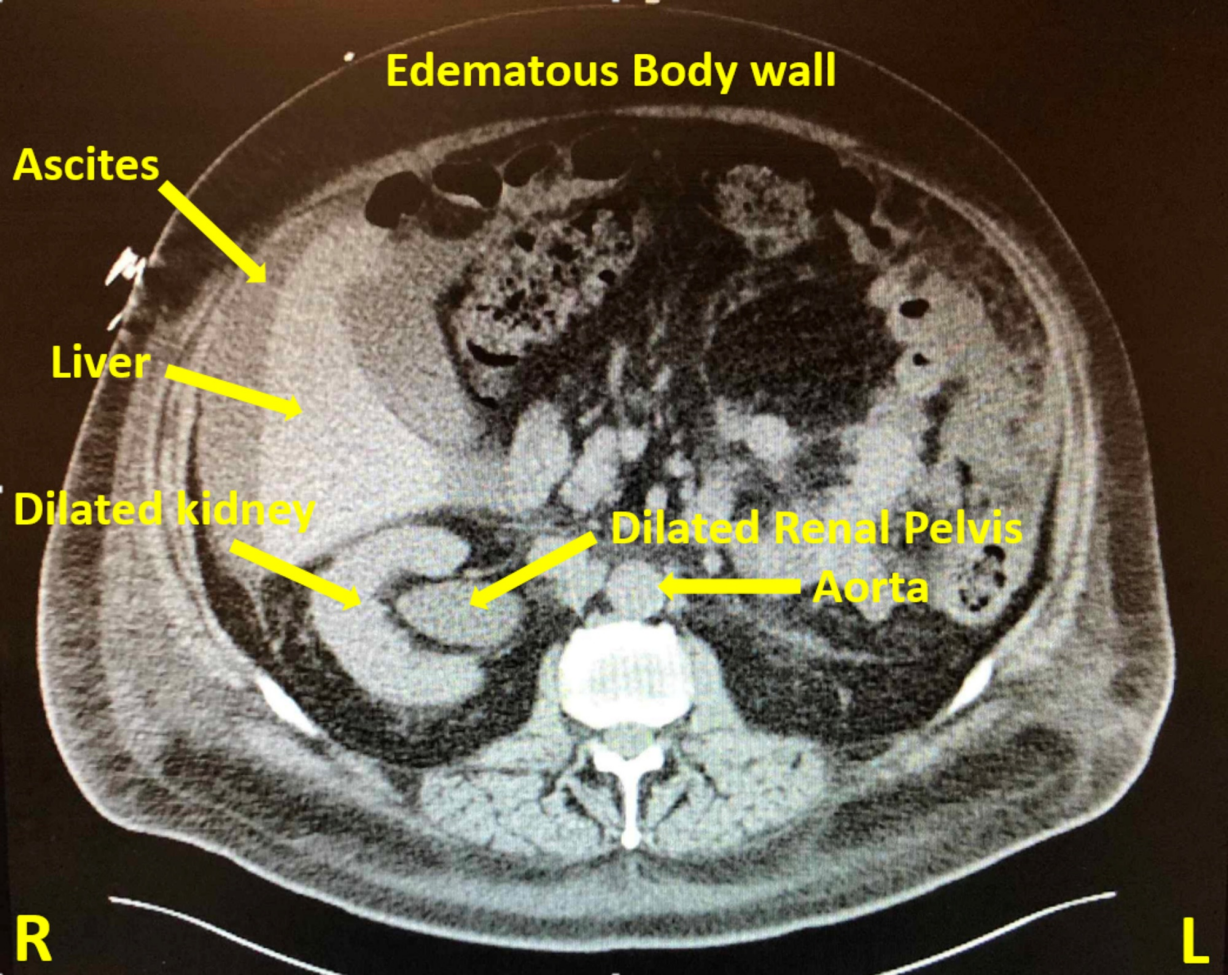
Computed tomography (CT) of the abdomen/pelvis with oral contrast The scan revealed moderate right hydro-uretero-nephrosis extending all the way to the uretero-vesicular junction; wall thickening along the superior and posterior margin of the bladder; moderate-large right pleural effusion; moderate abdominal and pelvic ascites; generalized anasarca of the soft tissues.

**Figure 3:**
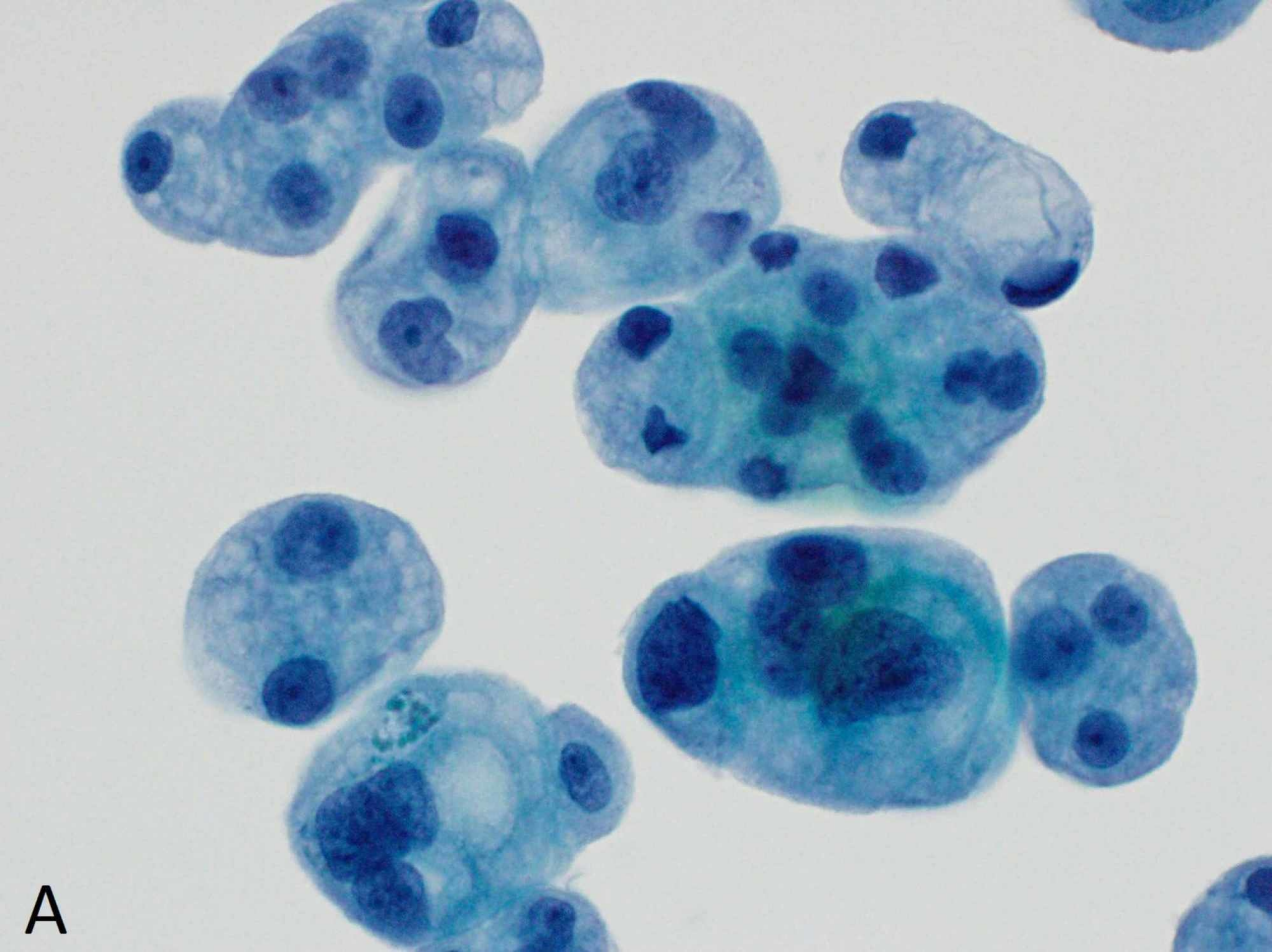
Pleural fluid cytology - ThinPrep monolayer preparation (Cytyc, Boxborough, MA, USA) Clusters of cytologically malignant epithelial cells are present (Papanicolaou stain x600).

**Figure 4:**
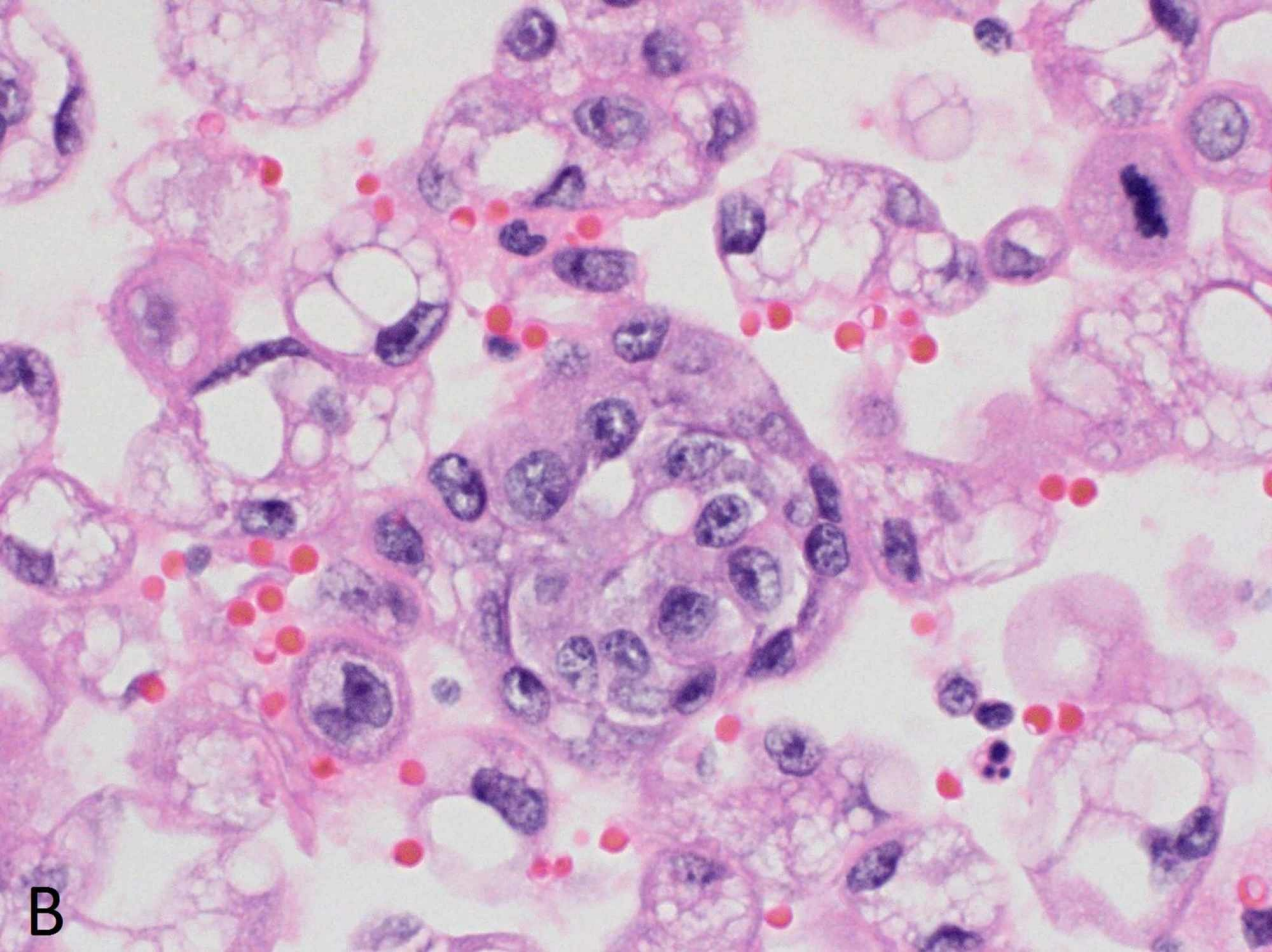
Pleural fluid cytology - cell block Clusters of malignant cells, many containing degenerative cytoplasmic vacuoles, are present (hematoxylin and eosin (H&E) stain x600).

**Figure 5:**
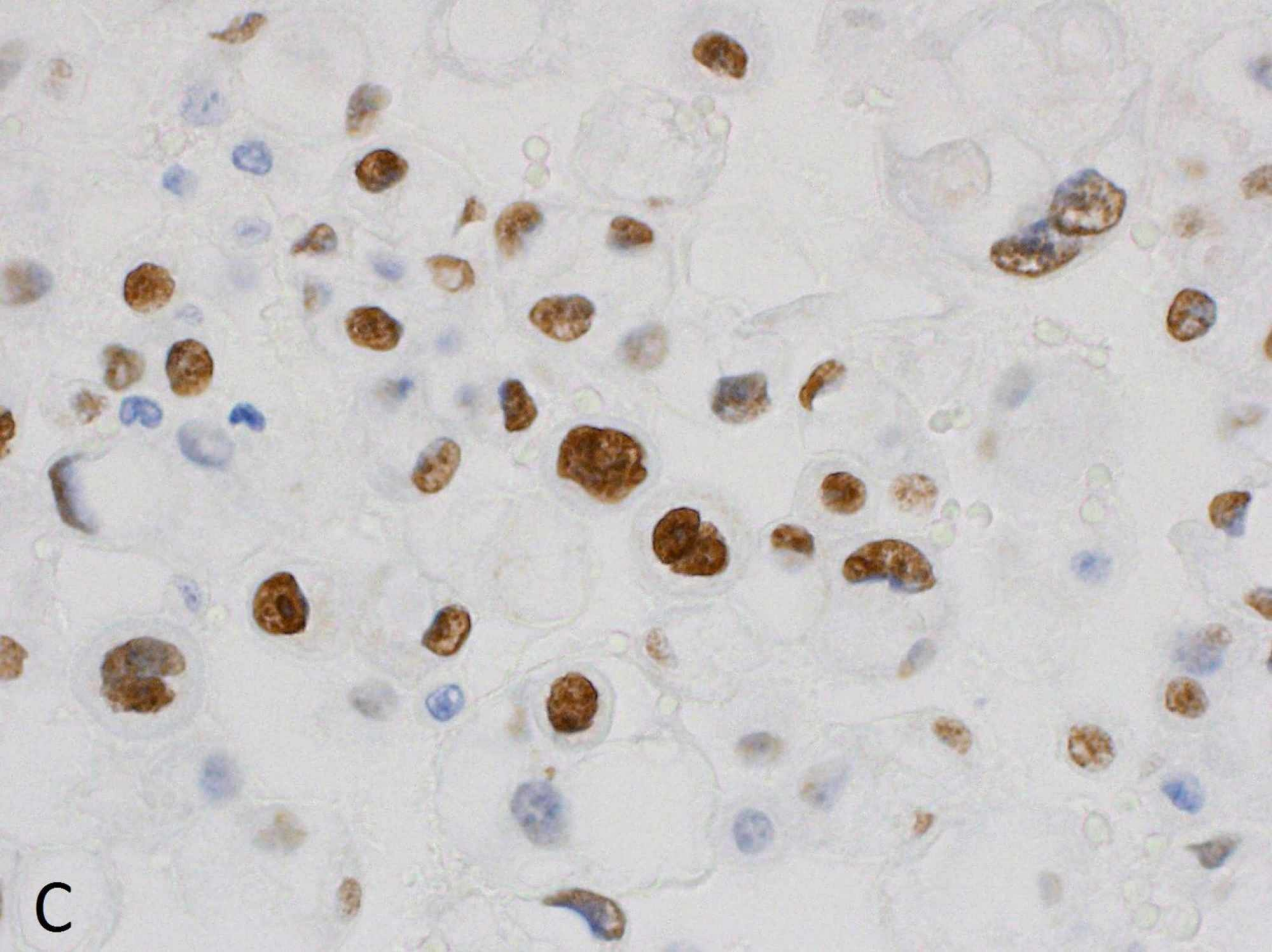
Pleural fluid cytology - cell block The cells show strong nuclear staining for GATA-3 (GATA-3 immunostain x600). The cells were also strongly positive for both cytokeratin 7 (CK7) and cytokeratin 20 (CK20). The CK7+, CK20+, GATA-3+ immunophenotypic profile is most consistent with urothelial carcinoma.

Within a week, he presented to the ED with recurrent right-sided pleural effusion, bloating and acute kidney injury. Computed tomography (CT) abdomen-pelvis at presentation showed right-sided hydroureteronephrosis extending down to the uretero-vesicular junction and an irregular bladder wall thickening concerning for primary bladder tumor (Figure 2). In the interim, the pleural fluid cytology had tested positive for malignant cells concerning for metastatic carcinoma of primary urothelial origin (Figures 3-5).

Urology, pulmonology, and oncology teams were involved in the case. Transurethral resection of bladder tumor (TURBT) was performed with removal of a 5-cm papillary, high grade appearing tumor along the posterior bladder wall. A double J ureteral stent was deployed on the right side as well. A pleurex catheter was inserted in the right pleural space and successful talc pleurodesis was performed post effusion drainage. The pathology report confirmed the diagnosis of invasive high-grade urothelial carcinoma invading the lamina propria.

Hematology-oncology unit planned for outpatient immunotherapy with Azetolizumab. Unfortunately, he was re-admitted directly from the infusion clinic for worsening acute kidney injury, hyperkalemia, and anasarca. He had a complicated hospital course that required high-level care including pressor support, mechanical ventilation, and renal replacement therapy. His clinical status continued to decline despite aggressive resuscitative efforts. He passed on day 36 from his initial presentation.

## Discussion

The proposed mechanism of the MPE includes infiltration of cancer cells into the pleural space via blood vessels or lymphatics, and/or passage of cancer cells from the peritoneal space into the pleural cavity through diaphragmatic pores [5]. There are different factors that predict prognosis. The late effects of normal tissue (LENT) score comprising of four factors (pleural fluid lactate dehydrogenase, Eastern Cooperative Oncology Group (ECOG) performance score, blood neutrophil-to-lymphocyte ratio, and tumor type) is a validated risk stratification tool [6]. MPEs arising from primary urological cancers predict a median survival of 33 days. This patient had a LENT score of 3 suggestive of moderate risk. MPEs usually have rapid re-accumulation eliciting symptoms such as dyspnea, cough, or chest pain. The median length of survival for patients who are diagnosed with MPEs is six months [7]. Treatment is mostly focused on palliative symptomatic relief aimed at effusion drainage and preventing reaccumulation. Different approaches can be employed in an appropriate setting and include intermittent thoracentesis, chemical pleurodesis, placement of an indwelling tunneled pleural catheter, pleurectomy, shunt, etc. [8].

## Conclusions

This case describes an unusual metastasis to the pleura in a patient with underlying urothelial carcinoma. There are only a few reported cases till date. Metastatic bladder cancer should be considered in an appropriate clinical setting in an atypical presentation.
